# Sox10^+^ adult stem cells contribute to biomaterial encapsulation and microvascularization

**DOI:** 10.1038/srep40295

**Published:** 2017-01-10

**Authors:** Dong Wang, Aijun Wang, Fan Wu, Xuefeng Qiu, Ye Li, Julia Chu, Wen-Chin Huang, Kang Xu, Xiaohua Gong, Song Li

**Affiliations:** 1 Department of Bioengineering, University of California, Berkeley, California 94720, USA; 2 School of Optometry and Vision Science Program, University of California, Berkeley, California 94720, USA; 3 Department of Bioengineering, University of California, Los Angeles, California 90095, USA; 4 Department of Surgery, University of California, Davis, Sacramento, California 95817, USA; 5 Department of Cardiovascular Surgery, Union Hospital, Tongji Medical School, Huazhong University of Science and Technology, Wuhan 430022, China; 6 Division of Neurobiology, Department of Molecular and Cell Biology, Helen Wills Neuroscience Institute, Howard Hughes Medical Institute, University of California, Berkeley, California 94720, USA; 7 Interdisciplinary Institute of Neuroscience and Technology, Zhejiang University, Hangzhou, Zhejiang 310016, China

## Abstract

Implanted biomaterials and biomedical devices generally induce foreign body reaction and end up with encapsulation by a dense avascular fibrous layer enriched in extracellular matrix. Fibroblasts/myofibroblasts are thought to be the major cell type involved in encapsulation, but it is unclear whether and how stem cells contribute to this process. Here we show, for the first time, that Sox10^+^ adult stem cells contribute to both encapsulation and microvessel formation. Sox10^+^ adult stem cells were found sparsely in the stroma of subcutaneous loose connective tissues. Upon subcutaneous biomaterial implantation, Sox10^+^ stem cells were activated and recruited to the biomaterial scaffold, and differentiated into fibroblasts and then myofibroblasts. This differentiation process from Sox10^+^ stem cells to myofibroblasts could be recapitulated *in vitro*. On the other hand, Sox10^+^ stem cells could differentiate into perivascular cells to stabilize newly formed microvessels. Sox10^+^ stem cells and endothelial cells in three-dimensional co-culture self-assembled into microvessels, and platelet-derived growth factor had chemotactic effect on Sox10^+^ stem cells. Transplanted Sox10^+^ stem cells differentiated into smooth muscle cells to stabilize functional microvessels. These findings demonstrate the critical role of adult stem cells in tissue remodeling and unravel the complexity of stem cell fate determination.

Biomaterials and biomedical devices have been widely used in clinical applications, such as drug releasing devices^1^,^2^, glucose sensors^3^, vascular grafts^4^,^5^, bone and cartilage grafts^6^,^7^. The implants generally induce foreign body reaction and end up with encapsulation with a thick avascular fibrous layer composed of dense extracellular matrix around implants^1^. Encapsulation can be detrimental to some medical devices including drug releasing systems and glucose sensors, which blocks the free movement of molecules between the devices and host tissues, thus impairs their performances. Much effort has been made to modify biomaterials to reduce capsular thickness and/or enhance angiogenesis for better integration into host tissues^1^,^8^,^9^,^10^. On the other hand, encapsulation could also be beneficial for some implants such as vascular, bone and cartilage grafts. Upon transplantation, host cells are recruited to the biomaterial grafts from surrounding tissues, form a capsule layer, and infiltrate into the grafts if they have a porous structure, which promotes their integration into host tissues^6^,^11^,^12^.

Although encapsulation is an important phenomenon, there are few studies on the cellular origins of encapsulation. Traditionally, fibroblasts are thought to be the major cells contributing to biomaterials encapsulation^1^. Encapsulation is a fibrosis process and it shares the common characteristics of organ fibrosis or scar formation: the accumulation of extracellular matrix and the degeneration of microvessels^13^. Most studies on cellular origins of fibrosis are from organ fibrosis^14^. For example, during skin wound healing and scar formation, fibroblasts are activated to proliferate, differentiate into myofibroblasts, secrete excessive extracellular matrix, express smooth muscle α-actin (ACTA2) and apply contractive force to close the wound^14^. Studies from organ fibrosis also reported that perivascular cells were another important source of myofibroblasts^15^,^16^,^17^,^18^,^19^. However, the relationship between fibroblasts and perivascular cells, and the involvement of other stem cells remain to be elucidated.

In recent years, several research groups including our laboratory reported that Sox10^+^ adult stem cells existed in multiple tissues including large blood vessels walls and surrounding tissues, which could be recruited to vascular graft contributing to tissue remodeling^5^,^20^. We sought to investigate whether and how Sox10^+^ stem cells participate in biomaterials encapsulation in general. Here we showed that Sox10^+^ adult stem cells were found in the stroma of subcutaneous loose connective tissues. We utilized *in vivo* subcutaneous biomaterial implantation, *in vitro* stem cell culture, *ex vivo* tissue explant culture, *in vivo* stem cell transplantation and two-photon live animal microscopy experiments and showed that Sox10^+^ adult stem cells differentiated into fibroblasts at early stages of biomaterial implantation, and then differentiated into myofibroblasts contributing to encapsulation, or perivascular cells supporting angiogenesis. The same cell population can give rise to both fibrotic/capsule tissues and microvessels depending on local microenvironment, which demonstrates the critical role of adult stem cells in tissue remodeling and unravels the complexity of stem cell fate determination.

## Results

### Sox10^+^ cells were found in the stroma of subcutaneous loose connective tissues and activated upon biomaterial implantation

**Figure 1:**
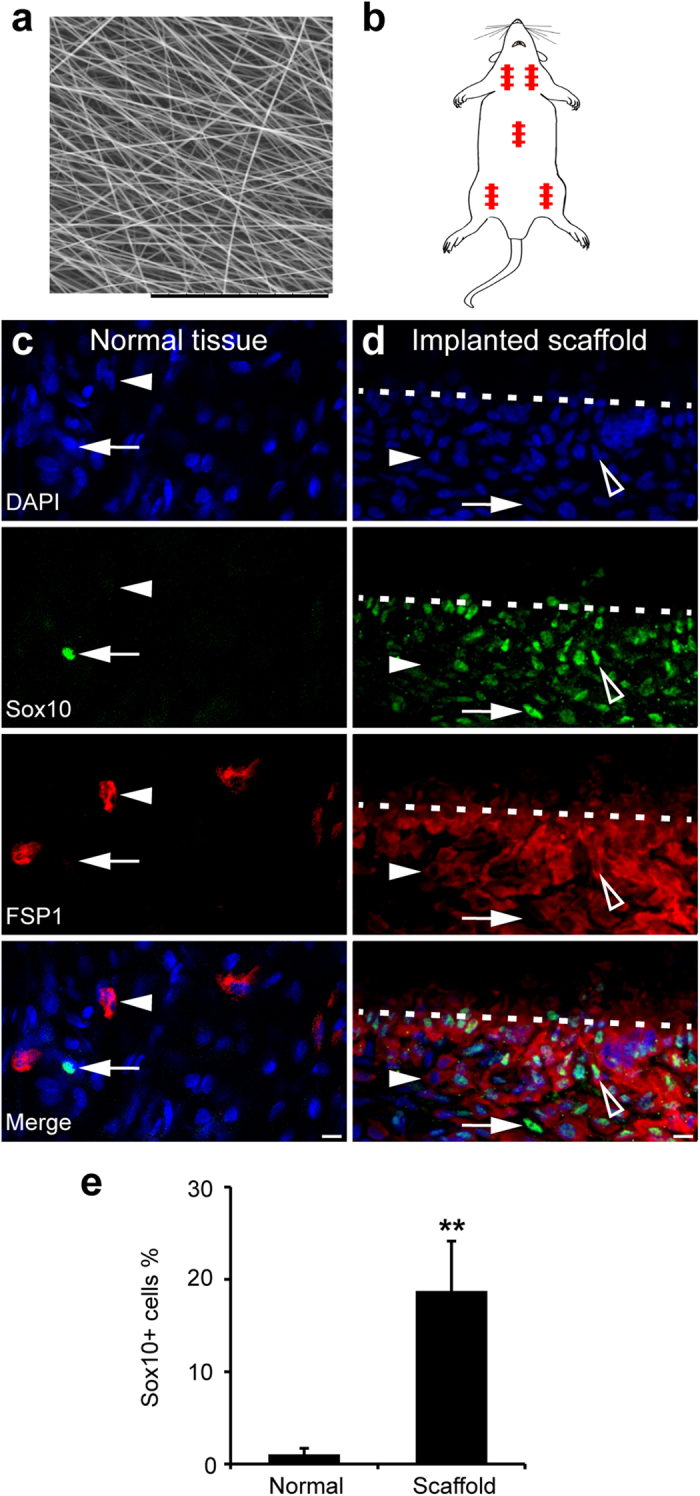
The activation and recruitment of Sox10^+^ cells after subcutaneous scaffold implantation. (**a**) Scanning Electron Microscopy image of the electrospun PLLA scaffold membrane. Scale bar, 100 μm. (**b**) Illustration of multiple subcutaneous scaffold implantation sites in a rat. (**c**,**d**) The cross sections of normal subcutaneous tissue (**c**) and implanted scaffold (**d**) were immunostained by the antibodies against stem cell marker Sox10 and fibroblast marker FSP1. Cell nuclei were stained by DAPI. Arrow, Sox10^+^ cells. Arrowhead, FSP1^+^ cells. Hollow arrowhead, Sox10^+^/FSP1^+^ cells. White dashed lines illustrate the boundary between the implanted scaffold and surrounding native tissue. Scale bar, 10 μm. (**e**) Percentages of Sox10^+^ cells before and after scaffold implantation were quantified. Mean ± SD of n = 3 independent experiments of n = 9 sections for each group. Student’s *t*-test was performed to analyze significant differences between groups. **P < 0.01.

To investigate whether Sox10^+^ adult stem cells contribute to biomaterial encapsulation, we prepared poly(L-lactic acid) (PLLA) scaffold membranes (Fig. 1a) and implanted them into multiple subcutaneous spaces of rats (Fig. 1b). Immunostaining showed sparse Sox10^+^ cells (<2%) and fibroblasts (~5%, expressing fibroblast-specific protein 1 (FSP1), a fibroblast marker) in the stroma of subcutaneous loose connective tissues before implantation (Fig. 1c; Figure S1). These Sox10^+^ cells did not express FSP1 (Fig. 1c), suggesting that they were a different stromal cell population from fibroblasts. One-week post-implantation, we found many Sox10^+^ cells (~20%) around the scaffold, and more than 90% of them showed FSP1 expression (Fig. 1d,e). We found similar phenomena in the scaffolds implanted into multiple subcutaneous sites of rats (Fig. 1b). These results are consistent with the traditional knowledge that fibroblasts are activated to proliferate upon injury. In addition, it demonstrates the connection between Sox10^+^ cells and fibroblasts *in vivo*.

### Sox10^+^ cells are a precursor of fibroblasts

**Figure 2:**
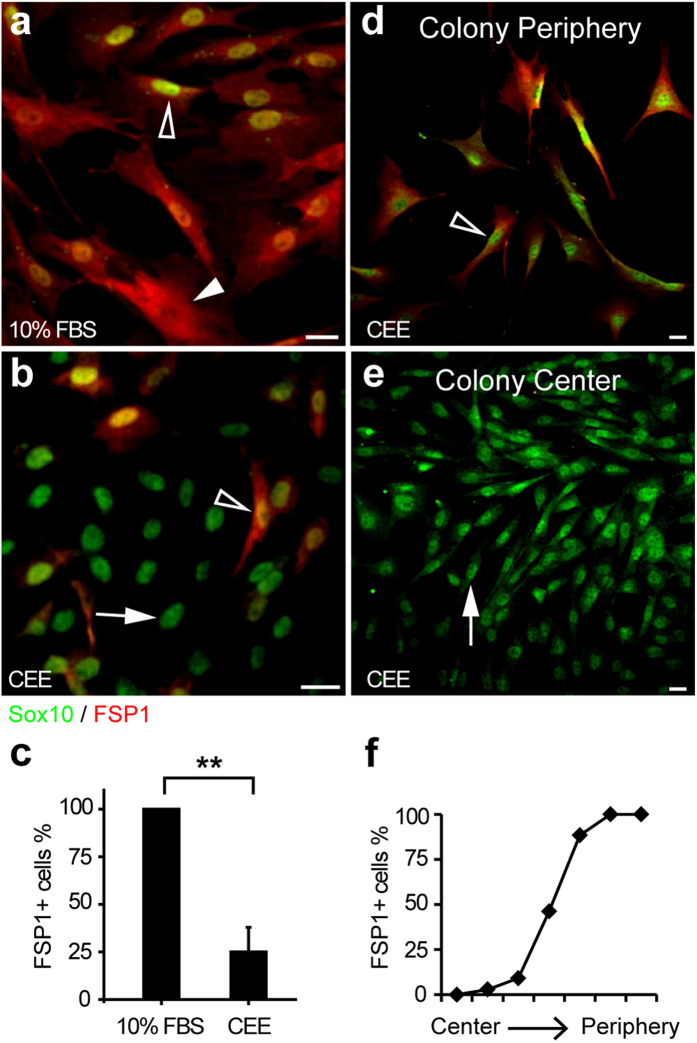
Sox10^+^ cells as a precursor of fibroblasts. (**a**,**b**) Primary cells from rat subcutaneous connective tissues were cultured in the medium of 10% FBS (**a**) and CEE (**b**) and immunostained with the antibodies against Sox10 and FSP1. (**c**) Percentages of FSP1^+^ cells among the primary cells in the media of 10% FBS and CEE. (**d**,**e**) The cells of a colony cultured in CEE medium were immunostained with the antibodies against Sox10 and FSP1. (**f**) Percentages of FSP1^+^ cells from the colony center to periphery. Arrow, Sox10^+^ cells. Arrowhead, FSP1^+^ cells. Hollow arrowhead, Sox10^+^/FSP1^+^ cells. Mean ± SD of n = 3 independent experiments of n = 9 fields for each group. Student’s *t*-test was performed to analyze significant differences between groups. **P < 0.01. Scale bar, 20 μm.

To determine the relationship of Sox10^+^ and FSP1^+^ cells, we performed conventional primary fibroblasts culture. Subcutaneous loose connective tissues were harvested from normal rats, cut into small pieces and put into culture dishes in the medium supplemented with 10% fetal bovine serum (FBS). The cells migrated from the tissues and proliferated with a classic fibroblast cell shape. After one week of culture, the primary cells were passaged to new dishes and used for analysis. By immunostaining, we found that most of the cells were Sox10^+^/FSP1^+^ (Fig. 2a; Figure S2a), similar to the cells around implanted scaffolds (Fig. 1d). Considering that high concentration of FBS may induce cell differentiation quickly, we switched to a custom-made chick embryo extract (CEE) medium. We obtained a Sox10^+^ population in CEE medium, among which about 25% of cells expressed FSP1 (Fig. 2b,c; Figure S2b), suggesting that CEE medium could slow down the differentiation of Sox10^+^ cells.

To isolate single Sox10^+^/FSP1^−^ cells from Sox10^+^/FSP1^+^ cells, we plated the mixed cell population at very low density (~100 cells per 100-mm dish), and found most of them could form colonies after 10-day culture in CEE medium (Figure S3). By immunostaining, we found that, some colonies consisted of all Sox10^+^/FSP1^+^ cells while others had all Sox10^+^ cells with gradient expression pattern of FSP1 as the lowest level in the center and the highest at the periphery (Fig. 2d–f; Figure S4). Mixture of these colonies were further passaged for additional analysis of single-cell colony formation in the medium with 10% FBS, which may induce spontaneous differentiation. We found that all colonies became FSP1^+^ cells with few Sox10^+^ ones after two passages (Figure S5), indicating that differentiated fibroblasts lost Sox10 expression. These results suggest that Sox10^+^ cells are a precursor of fibroblasts.

### Sox10^+^ cells are multipotent stem cells

**Figure 3:**
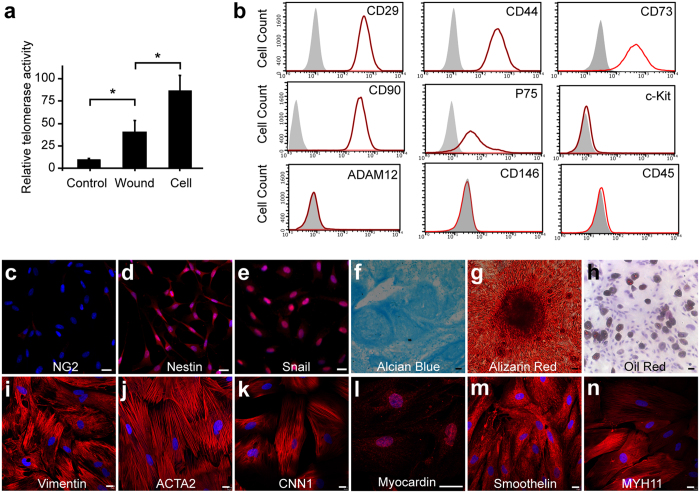
Multipotency of Sox10^+^ cells. (**a**) Telomerase activity assay was performed on the native subcutaneous tissue (Control, without injury), wounded tissue and primary cells. Mean ± SD of n = 3 independent experiments for each group. Student’s *t*-test was performed to analyze significant differences between groups. *P < 0.05. (**b**) Flow cytometry analysis of primary Sox10^+^ cells with the antibodies against CD29, CD44, CD73, CD90, P75, c-Kit, ADAM12, CD146 and CD45. (**c**–**e**) The primary Sox10^+^ cells were immunostained by the antibodies against NG2 (**c**), Nestin (**d**) and Snail (**e**). (**f**–**n**) After culture in specific differentiation media, the cells were stained by Alcian blue (**f**), Alizarin red (**g**), Oil red (**h**) and antibodies against Vimentin (**i**), ACTA2 (**j**), CNN1 (**k**), Myocardin (**l**), Smoothelin (**m**) and MYH11 (**n**). Cell nuclei were stained by DAPI. Scale bar, 20 μm.

We further characterized the stem cell properties of Sox10^+^ cells *in vitro*. By quantitative real-time polymerase chain reaction, we found the cultured cells had high telomerase activity compared to wounded and normal tissue (Fig. 3a). By flow cytometry and immunostaining (Fig. 3b–e), we found Sox10^+^ cells expressed markers of mesenchymal stem cells, such as CD29, CD44, CD73, CD90 and Snail. They also expressed neural crest stem cell markers, P75 and nestin, but not c-Kit, ADAM12, CD146 or NG2. They did not express CD45 indicating that they were not derived from hematopoietic lineage. In specific induction media, Sox10^+^ cells could differentiate into chondrogenic (Alcian blue), osteogenic (Alizarin red) and adipogenic (Oil red) cells (Fig. 3f–h). In the medium with 10% FBS and 10 ng/ml transforming growth factor β1 (TGFβ1), they differentiated into myofibroblasts (Vimentin^+^ and smooth muscle α-actin^+^/ACTA2^+^) and smooth muscle cells (SMCs) (ACTA2^+^, Calponin1/CNN1^+^, Myocardin^+^, Smoothelin^+^ and smooth muscle myosin heavy chain^+^/MYH11^+^) (Fig. 3i–n).

Taken together, these results suggest that there are Sox10^+^ adult stem cells in the stroma of subcutaneous loose connective tissues, which can be activated to proliferate and differentiate into fibroblasts at early stages of biomaterial implantation. They also have the potential to differentiate into myofibroblasts and SMCs *in vitro*.

### Sox10^+^ adult stem cells contribute to both encapsulation and microvascularization after transplantation *in vivo*

**Figure 4:**
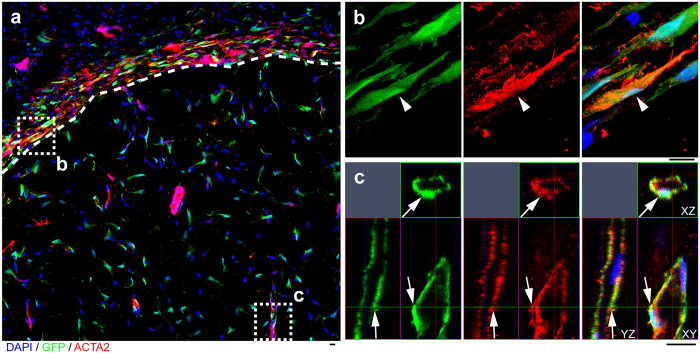
Sox10^+^ adult stem cells contribute to both encapsulation and microvascularization. (**a**) The cross section of the Matrigel plug with GFP^+^ cells was immunostained by the antibody against ACTA2. Cell nuclei were stained by DAPI. The dashed curve outlines the capsule layer around the Matrigel plug. (**b**,**c**) The zoomed-in images of the two dashed squares in a (**c**, orthogonal view). Scale bar, 10 μm.

To further investigate Sox10^+^ adult stem cell fate *in vivo*, we performed stem cell transplantation experiments. Our flow cytometry results showed that Sox10^+^ stem cells also expressed P75, which is a surface marker for neural crest stem cells and can be conveniently used for live cell sorting. Thus we used P75 to further purify the population for the following experiments. The green fluorescent protein (GFP)^+^/Sox10^+^/P75^+^ cells were isolated from the first passage of Sox10^+^ cells from the subcutaneous loose connective tissues of GFP-rats using P75 as the marker for magnetic cell separation, and were subsequently transplanted into the subcutaneous space of immunodeficient mice by Matrigel plug assay. After two weeks, the Matrigel plugs were harvested for immunohistology. We found that, in the periphery of the Matrigel plug, the GFP^+^ cells were elongated, aligned parallel to each other, had strong ACTA2 signal, and formed a capsule layer wrapping around the Matrigel plug, indicating that they had differentiated into myofibroblasts (Fig. 4a and b; Figure S6). It was a surprise that, in the center of the Matrigel plug, the GFP^+^ cells formed tubular structures and also expressed ACTA2, suggesting that they might have differentiated into perivascular cells (Fig. 4a and c; Figure S6).

**Figure 5:**
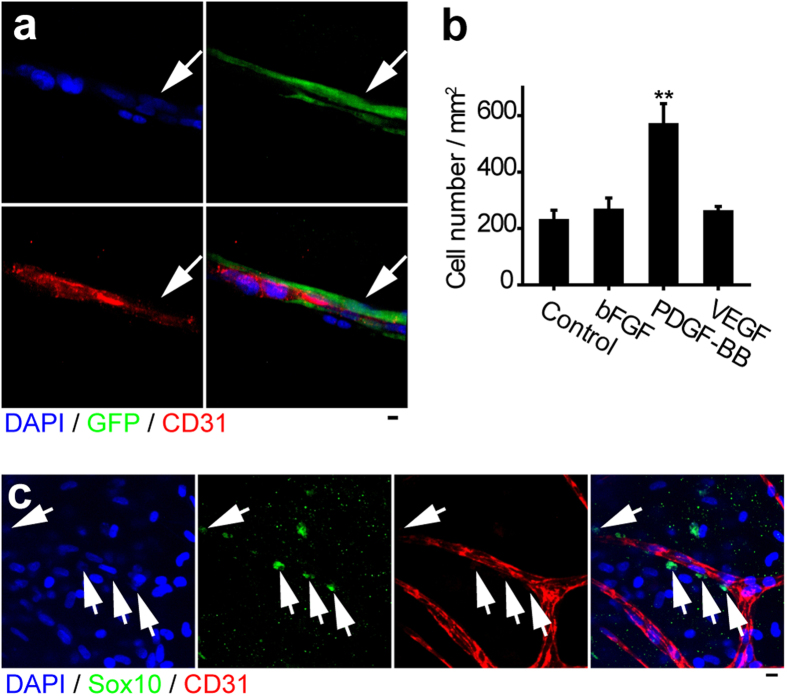
Sox10^+^ cells contribute to microvessel formation *in vitro*. (**a**) GFP^+^/Sox10^+^/P75^+^ stem cells and vascular endothelial cells were co-cultured on Matrigel and immunostained by the antibody against CD31. Arrow, GFP^+^ cells. (**b**) Chemotaxis assay (transwell cell migration) of GFP^+^/Sox10^+^/P75^+^ stem cells to bFGF, PDGF-BB and VEGF. Mean ± SD of n = 3 independent experiments of n = 9 wells for each group. Student’s *t*-test was performed to analyze significant differences between groups. **P < 0.01. (**c**) *Ex vivo* tissue explant culture sample was stained by the antibodies against Sox10 and CD31. Arrow, Sox10^+^ cells. Cell nuclei were stained by DAPI. Scale bar, 10 μm.

To further examine the role of Sox10^+^ stem cells in vascular regeneration, we performed Matrigel tube formation assay and found GFP^+^/Sox10^+^/P75^+^ stem cells formed microvessels when co-cultured with vascular endothelial cells (Fig. 5a). By transwell cell migration assay, we found that they had a chemotactic response to platelet-derived growth factor BB (PDGF-BB), an important growth factor involved in microvessel formation (Fig. 5b). In an *ex vivo* three-dimensional tissue explant culture model, Sox10^+^ cells co-migrated with vascular endothelial cells and newly sprouting microvessels (Fig. 5c). Thus, *in vivo, in vitro* and *ex vivo* results showed that Sox10^+^ adult stem cells contributed to both encapsulation and microvascularization.

**Figure 6:**
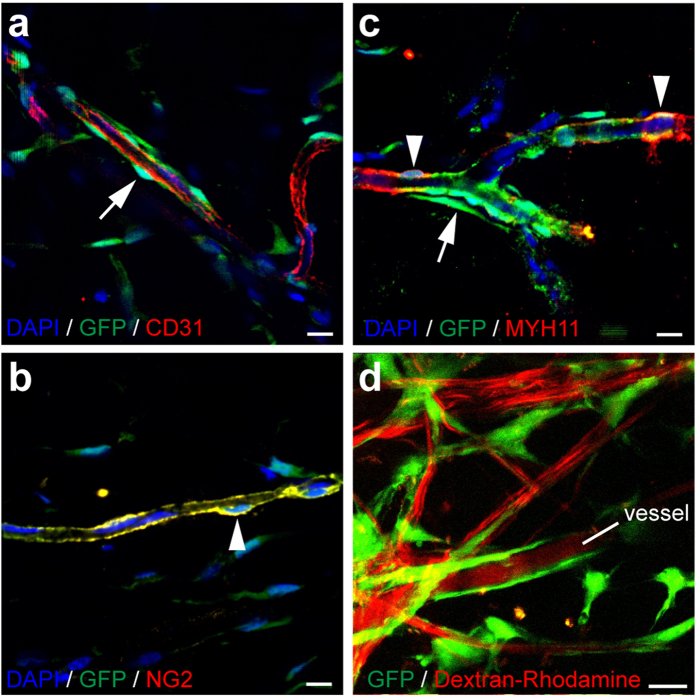
Sox10^+^ adult stem cells contribute to functional microvessels. (**a**–**c**) The cross sections of Matrigel plug with GFP^+^ cells were immunostained with the antibodies against CD31 (**a**), NG2 (**b**) and MYH11 (**c**). (**d**) Two-photon image of mouse dorsal skinfold chamber transplanted with GFP^+^/Sox10^+^ stem cells. Dextran-Rhodamine was injected into the mouse through tail vein before imaging. Cell nuclei were stained by DAPI. Arrow, GFP^+^ cells. Arrowhead, double positive cells. Scale bar, 10 μm.

We also examined other vascular markers of GFP^+^ vessels in the center of Matrigel plug and found GFP^+^ mural cells wrapped around CD31^+^ endothelial cells, expressed NG2 and MYH11 (Fig. 6a–c; Figure S7). It was interesting that these GFP^+^ microvessels had uniform expression of ACTA2 and NG2, but had a mosaic pattern of MYH11 expression (Figs 4 and 6; Figure S7), suggesting that they were in the process of maturation into SMCs. To examine whether the microvessels formed by GFP^+^/Sox10^+^ stem cells are functional, we implanted them into the skinfold chamber of immunodeficient mice (Figure S8) and imaged them by two-photon microscopy. By tail vein injection of Dextran-Rhodamine, we found the microvessels formed by GFP^+^ cells were perfused with the dye and thus were functional (Fig. 6d).

### Fibroblast as an intermediate cell type in the process of perivascular cell maturation

**Figure 7:**
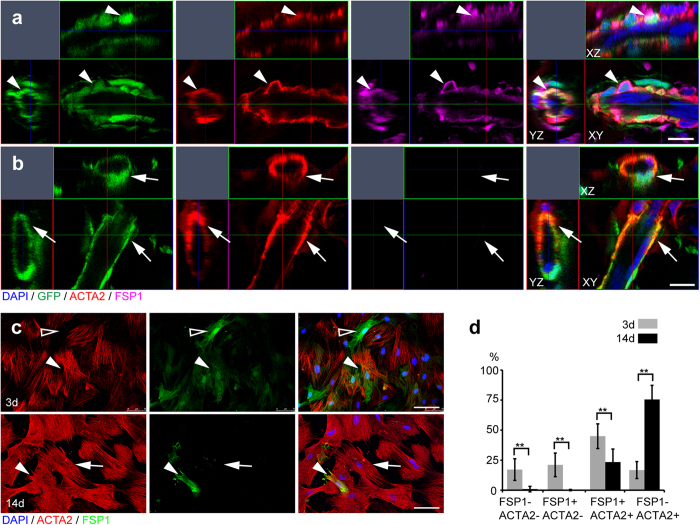
FSP1 expression during microvessel formation. (**a**,**b**) Orthogonal views of the cross sections of the Matrigel plug with GFP^+^ cells, which were immunostained by the antibodies against ACTA2 and FSP1. Scale bar, 10 μm. Arrowhead, GFP^+^/ACTA2^+^/FSP1^+^ cells. Arrow, GFP^+^/ACTA2^+^ cells. (**c**) Sox10^+^ stem cells were cultured in the medium with 10% FBS and 10 ng/ml TGFβ1 for 3 and 14 days, and immunostained by antibodies against ACTA2 and FSP1. Cell nuclei were stained by DAPI. Scale bar, 100 μm. Hollow arrowhead, FSP1^+^ cells. Arrowhead, ACTA2^+^/FSP1^+^ cells. Arrow, ACTA2^+^ cells. (**d**) Percentages of different cell populations were quantified. Mean ± SD of n = 3 independent experiments of n = 12 fields for each group. Student’s *t*-test was performed to analyze significant differences between groups. **P < 0.01.

To investigate the cell fate decision between fibroblasts and vascular mural cells, we performed immunostaining of GFP^+^ microvessels in the Matrigel plug. We found that the GFP^+^ cells lost Sox10 expression two-week post-implantation. There were GFP^+^/ACTA2^+^/FSP1^+^ mural cells in some microvessels (Fig. 7a), but some GFP^+^/ACTA2^+^ mural cells already lost FSP1 expression (Fig. 7b). To investigate the temporal dynamics of FSP1 expression, we induced Sox10^+^ stem cells to differentiate into SMCs *in vitro* as mentioned before (Fig. 3i–n). We found that most of the cells expressed FSP1 with or without ACTA2 expression (FSP1^+^/ACTA2^−^, or FSP1^+^/ACTA2^+^) at early stages (3 days), however at late stages (14 days), most of the cells were FSP1^−^/ACTA2^+^ and only a few FSP1^+^ cells remained (Fig. 7c,d). These results suggest that FSP1^+^ mural cells may be an intermediate cell type during perivascular cell maturation.

## Discussion

Previous studies show that Sox10 is a marker of neural crest cells during embryonic development, which give rise to most craniofacial tissues including connective tissues and vascular SMCs^21^,^22^. In recent years, Sox10^+^ stem cells have been reported to exist in multiple adult tissues, including inferior turbinate^23^, periodontal ligament^24^, hair follicle^25^,^26^, mammary epithelium^27^, and bone marrow^28^. Sox10 could also promote tumor formation^29^. In our previous work, Sox10^+^ adult stem cells were found in large blood vessel walls and surrounding tissues, and were recruited to vascular grafts contributing to vascular remodeling^5^,^20^,^30^. In this study, we further demonstrate that Sox10^+^ stem cells have a more universal distribution and can be found in the stroma of subcutaneous loose connective tissues, which could contribute to both encapsulation/fibrosis and microvascularization. Our studies, together with others, indicate that Sox10^+^ stem cells play important roles in adult tissue remodeling.

Although biomedical devices have been widely used in clinical applications, there remains a high failure rate^9^. Encapsulation is one of the major factors that cause the failure of implanted devices^1^,^9^. Most research groups have been focusing on modifying biomaterial composition, surface, structure or morphology to reduce capsule thickness and/or enhance angiogenesis^1^,^9^,^10^. On the contrary, some other research groups harnessed biomaterial encapsulation to make tubular structures composed of fibrous capsule tissues as vascular grafts^31^. Given the importance of biomaterial encapsulation, however, little attention has been paid on its cellular origins. Traditionally, fibroblasts are thought to be the major cells contributing to encapsulation, which is similar to skin wound healing and scar formation^1^,^14^. In this study, we found that Sox10^+^ adult stem cells in the stroma of subcutaneous loose connective tissues are a precursor of proliferating fibroblasts and myofibroblasts during encapsulation. Moreover, Sox10^+^ adult stem cells can contribute to microvascularization by differentiating into perivascular cells (Fig. 4). To our knowledge, this is the first report that a type of adult stem cells can directly contribute to both encapsulation/fibrosis and microvascularization.

Myofibroblasts and vascular SMCs are similar as they both express ACTA2 and produce contractile force^14^,^32^. A distinction is their tissue locations, as myofibroblasts usually exist in dense connective tissues enriched in extracellular matrix, while vascular SMCs wrap around blood vessels. It was reported that the ACTA2 gene expression was regulated differently in these two types of cells^33^. Some markers are used to separate these two cell types, such as MYH11 and Smoothelin, which are expressed in SMCs, but not in myofibroblasts^34^,^35^. However, it was also reported that differentiating myofibroblasts could express SMC markers CNN1, Smoothelin and MYH11^36^. Our study suggests that Sox10^+^ stem cells may go through a common fibroblast-like stage and further differentiate into myofibroblasts or SMCs, depending on the microenvironment.

At present, fibroblasts have not been well defined and previous observation and concept of fibroblasts might include cells at various stages of differentiation as they appear quite heterogeneous. They are mostly described based on cell morphology and their tissue location: they are embedded in the stroma of connective tissues, have a spindle shape, and are not vascular, epithelial or inflammatory cells^37^,^38^. A few markers have been proposed for identifying fibroblasts, including FSP1. It was first reported as a fibroblast marker by Strutz *et al*. and was found to be able to distinguish fibroblasts from epithelial cells^39^. FSP1 has also been used as an early marker for epithelial-mesenchymal transition and may play important roles during fibrosis^39^,^40^,^41^,^42^. Arteriole SMCs were found to express FSP1 in benign nephrosclerosis samples and thought to contribute to fibrosis process^43^. In contrast to the traditional opinion that FSP1 expression is indicative of fibrosis, our results showed that FSP1^+^ cells still retain the capability of forming microvessels. This result is consistent with the reports that FSP1 protein could promote angiogenesis during tumor formation^44^,^45^,^46^ and fibroblasts could stabilize *in vitro* engineered microvessels^47^. In our stem cell transplantation experiment *in vivo*, GFP^+^/Sox10^+^ stem cells differentiated into two types of perivascular cells: FSP1^+^/ACTA2^+^ and FSP1^−^/ACTA2^+^. During Sox10^+^ stem cells’ differentiation into SMCs *in vitro*, FSP1 expression was decreased in the late stages. These results indicate that FSP1 might be an intermediate stage marker during the differentiation of Sox10^+^ stem cells into perivascular cells or SMCs.

As encapsulation/fibrosis and microvascularization lead to distinct clinical outcomes, understanding the underlying regulatory mechanisms is of great importance. These two processes seem to compete with each other during tissue regeneration, as encapsulation/fibrosis is usually accompanied by microvascular degeneration^1^,^13^. However, there is evidence that the cells involved in these two processes may be related. For example, pericytes or perivascular progenitor cells^17^,^18^,^19^ and SMCs^43^ have been reported to give rise to myofibroblasts during fibrosis. On the other hand, there has also speculation that myofibroblasts, when put in the right microenvironment, such as the media layer of large blood vessel walls, might differentiate into SMCs^35^, suggesting the possibility of reciprocal transformation of the two processes. Our study showed that even the cells expressing FSP1, a traditionally recognized fibroblast/fibrosis marker, could form microvessels. The microenvironment of the cells (in this study, cell location relative to a Matrigel plug) probably played a key role in cell fate determination.

In conclusion, this study provides a novel mechanism that Sox10^+^ adult stem cells in the stroma of subcutaneous loose connective tissues are a common precursor of fibroblasts/myofibroblasts and perivascular cells. These adult stem cells can first differentiate into fibroblast-like cells at early stages of biomaterials implantation, and then into myofibroblasts promoting encapsulation/fibrosis, or perivascular cells supporting microvessels. This study is focused on Sox10^+^ adult stem cells and their functions in encapsulation/fibrosis and microvessel regeneration. Indeed, this complex remodeling process also involves other cell types such as inflammatory, microvascular and other types of stem cells. Further studies are warranted to investigate how different cells, extracellular matrices as well as other microenvironmental factors interact with each other to determine cell fate in tissue remodeling.

## Materials and Methods

### Fabrication of biomaterial scaffold membrane

Microfiber scaffold membranes were fabricated by using PLLA (MW 67,400, Sigma Aldrich) as previously reported^5^,^48^. The polymer was completely dissolved in 1,1,1,3,3,3-hexafluoro-2-propanol (HFIP, Aladdin) at the concentration of 19% (w/v). Microfibrous scaffold were made by electrospinning. The negative voltage of 4.5 kV was applied to the collecting plate, and a positive voltage of 4 kV was applied to the spinneret by using a high-voltage generator (Gamma High Voltage, Ormond Beach, FL). The electrospinning process was allowed to proceed until an approximately 200 μm wall thickness was achieved. The structure of the scaffolds was characterized by using a scanning electron microscope (Hitachi TM-1000).

### Animal experiments

All the animal procedures in this study followed the National Institutes of Health guide for the care and use of Laboratory animals and were approved by the University of California Berkeley Animal Care and Use Committee. For rats, anesthesia was induced with 3% isoflurane in an induction chamber followed by subcutaneous injection of 0.05 mg/kg buprenorphine and 1 mg/kg meloxicam for analgesia. For mice, anesthesia was induced with 3% isoflurane in an induction chamber followed by subcutaneous injection of 0.1 mg/kg buprenorphine and 5 mg/kg meloxicam for analgesia. Maintenance anesthesia was with 1.5–2% isoflurane delivered by mask. Toe pinch was performed to confirm the anesthetic depth. Aseptic surgeries were performed in all the procedures. Post-surgery buprenorphine was given to the animals for analgesia. The body weight, appearance and appetite of the animals will be monitored every day. The animals were euthanized by CO_2_ inhalation until they ceased breathing completely and then followed by bilateral thoracotomy for rats, or cervical dislocation for mice. The samples were then collected for analysis.

Subcutaneous biomaterials implantation: Male Sprague Dawley (SD) rats with the body weight of about 100–150 g were used in the experiments. Small pieces of PLLA membrane (about 5 × 5 mm) were implanted into various subcutaneous spaces, such as abdomen, neck, groin and back. Normal SD rats of the same body weight were used as a control group. Each group had three animals.

Matrigel plug assay: Sox10^+^ stem cells were isolated from GFP-rats (Rat Resource & Research Center, # 0065), and the first passage of cells were used for isolating GFP^+^/Sox10^+^/P75^+^ cells by P75 magnetic cell separation (MACS kit, Miltenyi Biotec, 130-097-127) according to the product instructions. The GFP^+^/Sox10^+^/P75^+^ cells were then mixed with Matrigel (Corning) with the cell density of 2 × 10^6^/ml and then injected to immunodeficient NSG mice (The Jackson Laboratory, # 005557) subcutaneously. After two weeks, the Matrigel plugs were harvested for histological analysis.

Mouse dorsal skinfold chamber: The procedure for implanting dorsal skinfold chamber was described previously^49^, with minor modifications. The GFP^+^/Sox10^+^/P75^+^ stem cells prepared as mentioned above were mixed with Matrigel and injected into the skinfold chamber of NSG mice by a curved 30 G needle. For two-photon microscopy, the mice were anesthetized by isoflurane and placed on a custom-made stage for imaging. One hour before imaging, dextran-Rhodamine was injected to the mice through tail vein. The z-series images were taken at the wavelength of 890 nm.

### Cell culture, telomerase activity and differentiation

Sox10^+^ adult stem cells were isolated from subcutaneous loose connective tissue of SD rats or transgenic GFP-rats. Subcutaneous loose connective tissues were dissected and cut into small pieces and placed onto the culture dish coated with 1% CellStart (Invitrogen) to allow cells to migrate out of the tissue explants. The culture was maintained in Dulbecco’s Modified Eagle Medium (DMEM) supplemented with 10% FBS, or in an optimized CEE medium, which is composed of DMEM supplemented with 2% CEE (MP Biomedical), 2% FBS, 1% N2 (Invitrogen), 2% B27 (Invitrogen), 100 nM retinoic acid (Sigma-Aldrich), 50 nM 2-mercaptoethanol (Sigma-Aldrich), 1% Penicillin/Streptomycin and 20 ng/ml bFGF (R&D Systems) based on a previous established protocol^19^. Telomerase activity was performed by a Quantitative Telomerase Detection kit (US Biomax) according to the manufacturer instructions. For differentiation into fibroblasts, the cells were cultured in DMEM supplemented with 10% FBS. For differentiation into myofibroblasts and SMCs, the cells were cultured in DMEM supplemented with 10% FBS and 10 ng/ml TGFβ1 (Peprotech) for two weeks. For differentiation into chondrogenic, osteogenic and adipogenic cells, the cells were cultured in specific media as described previously^20^,^50^.

### Flow cytometry analysis

For flow cytometry analysis, the Sox10^+^ stem cells were detached by 0.2% ethylenediaminetetraacetic acid (EDTA) for about 10 min, and blocked in 1% bovine serum albumin (BSA) for 30 min on ice. The following primary antibodies were used: CD29 (555005, BD), CD44 (550974, BD), CD73 (551123, BD), CD90 (554898, BD), P75 (ab8874, Abcam), c-Kit (sc-1494, Santa Cruz Tech), CD146 (FAB3250F, R&D), CD45 (561867, BD), ADAM12 (14139-1-AP, Proteintech Group Inc.). For unconjugated primary antibodies, corresponding secondary antibodies were used. Isotype IgG was used as negative control. Propidium iodide was used to exclude dead cells. A Guava easyCyte™ Flow Cytometer was used for the analysis.

### Single-cell colony formation assay

Sox10^+^ cells were detached and suspended in CEE medium. About 100 cells were added to each 100-mm dish in CEE medium. Medium was changed every three days. For secondary and tertiary single-cell colony formation in the medium of 10% FBS, the cells of single colonies were first washed with phosphate buffered saline (PBS), 8-mm glass cylinders with grease (Dow Corning 976 V High-Vacuum grease) on the bottom side were placed around the colonies, and TrypLE (Invitrogen) was added into the cylinders to detach the cells. The mixture of single colonies were passaged to new dishes at the density of about 100 cells per 100-mm dish in the medium with 10% FBS.

### Transwell cell migration assay

24-well transwell plates (Costar, pore size: 8 μm) were used for the cell migration assay. For each well, 4 × 10^4^ GFP^+^/Sox10^+^/P75^+^ stem cells isolated as mentioned above were seeded onto the top insert for 2 h in DMEM supplemented with 0.5% BSA. The top inserts were then transferred to the wells with DMEM supplemented with 0.5% BSA (control) and specific growth factors: 10 ng/ml basic fibroblast growth factor (bFGF), platelet-derived growth factor BB (PDGF-BB) and vascular endothelial growth factor (VEGF). After 5 h, the cells were fixed, stained by DAPI and the migrating cells numbers were counted by confocal microscopy. There were three repeats for each group.

### Matrigel tube formation assay

GFP^+^/Sox10^+^/P75^+^ stem cells isolated as mentioned above were mixed with human microvascular endothelial cells (HMECs) (ratio: 0.3:1) and seeded onto the surface of Matrigel (Corning). The cells were fixed for immunostaining after overnight culture in EGM2 medium (Lonza).

### *Ex vivo* tissue explant culture models

Rat subcutaneous connective tissue was cut into small pieces and placed in collagen I (1 mg/ml, Millipore) gel. The culture medium was Opti-MEM (Invitrogen) supplemented with 20 ng/ml bFGF.

### Histology and immunostaining

Rat and mouse tissues and biomaterial samples were collected and fixed in 4% paraformaldehyde for half hour at room temperature. The animal tissue samples were then transferred to 15% and 30% sucrose overnight in cold room, and embedded in OCT for cryosection. For immunostaining, the cells were fixed with 4% paraformaldehyde for 15 min, the tissue sections were re-fixed with 4% paraformaldehyde for 10 min, before permeabilized in 0.5% Triton X-100 (Sigma-Aldrich) for 5 min. The samples were then blocked with 5% donkey serum for 1 h, incubated in primary antibodies for 2 h at room temperature, or overnight at cold room. The following primary antibodies were used: MYH11 (BT-562, Biomedical Technologies), CD31 (ab28364, abcam), Sox10 (sc-17342, Santa Cruz Biotech), Snail (sc-28199, Santa Cruz Tech), Tuj1 (MAB1637, EMD Millipore), NG2 (AB5320, EMD Millipore), ACTA2 (ab32575, abcam), FSP1 (ab27957, abcam), Vimentin (ab92547, abcam), Calponin1 (CNN1, ab46794, abcam), Smoothelin (sc-28562, Santa Cruz Tech), Myocardin (sc-34238, Santa Cruz Tech). After primary antibodies incubation, the samples were washed with 0.1% Triton for three times, incubated with corresponding AF488, 546 or 633-labeled secondary antibodies (Molecular Probes) and 4′,6-diamidino-2-phenylindole (DAPI). Fluorescence images were taken by confocal microscopy (Prairie, Zeiss LSM710 and Leica SP5-Blue). Nine cross sections were used to count Sox10^+^ cell number in normal tissue and around scaffold. For counting FSP1^+^ and ACTA2^+^ cells cultured in the media of CEE, 10% FBS and 10% FBS plus TGFβ1, at least nine fields of view were collected for each group. The figures were processed by Adobe Photoshop and Adobe Illustrator.

### Statistical analysis

Data were reported as means ± SD, unless otherwise indicated. All experiments were repeated at least three times. Student’s *t*-test was used for the analysis of differences between different groups. Significance level was set as *P* < 0.05 or 0.01.

## Additional Information

**How to cite this article**: Wang, D. *et al*. Sox10^+^ adult stem cells contribute to biomaterial encapsulation and microvascularization. *Sci. Rep.* **7**, 40295; doi: 10.1038/srep40295 (2017).

**Publisher's note:** Springer Nature remains neutral with regard to jurisdictional claims in published maps and institutional affiliations.

## Supplementary Material

Supplementary Figures
