# Supplementary material for: Sox10+ adult stem cells contribute to biomaterial encapsulation and microvascularization
Source: Sci Rep. 2017 Jan 10;7:40295. doi: 10.1038/srep40295 (PMC5223127; doi:10.1038/srep40295)
Supplement: Supplementary Figures [file srep40295-s1.pdf]

Sox10$^+$ adult stem cells contribute to biomaterial encapsulation and microvascularization

Dong Wang[1,2,3,†], Aijun Wang[1,4,†], Fan Wu[1], Xuefeng Qiu[1,3,5], Ye Li[6,7], Julia Chu[1], Wen-Chin Huang[1], Kang Xu[1,3], Xiaohua Gong[2] & Song Li[1,3,*]

[1]Department of Bioengineering, [2]School of Optometry and Vision Science Program, University of California, Berkeley, California 94720, USA; [3]Department of Bioengineering, University of California, Los Angeles, California 90095, USA; [4]Department of Surgery, University of California, Davis, Sacramento, California 95817, USA; [5]Department of Cardiovascular Surgery, Union Hospital, Tongji Medical School, Huazhong University of Science and Technology, Wuhan 430022, China; [6]Division of Neurobiology, Department of Molecular and Cell Biology, Helen Wills Neuroscience Institute, Howard Hughes Medical Institute, University of California, Berkeley, California 94720, USA; [7]Interdisciplinary Institute of Neuroscience and Technology, Zhejiang University, Hangzhou, Zhejiang 310016, China.

[†]These authors contributed equally to this work.

*Corresponding to: Song Li, Ph.D., Department of Bioengineering, University of California, Los Angeles, 5121 Engineering V, Los Angeles, CA 90095. Email: songli@ucla.edu

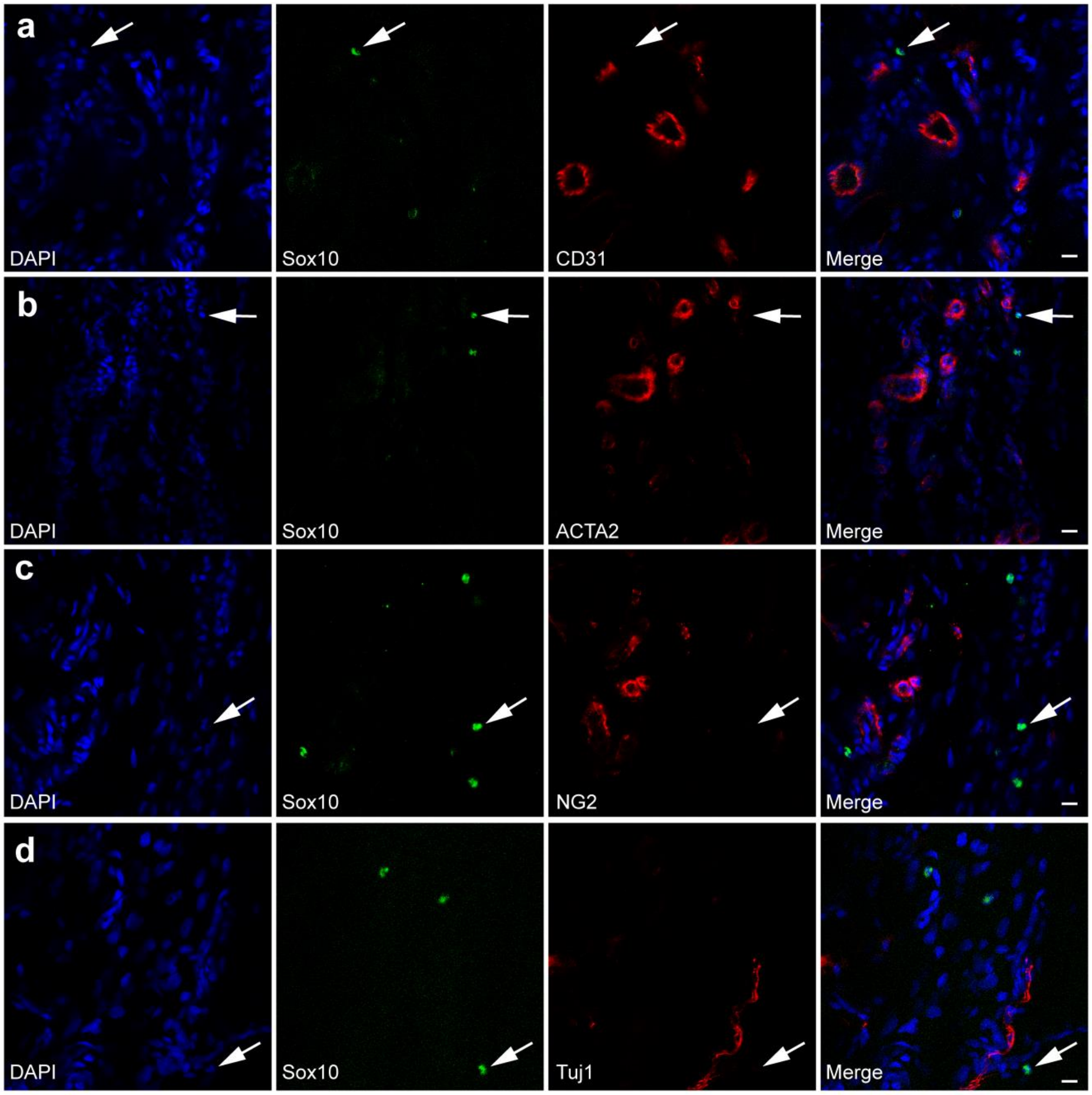

Figure S1. Immunohistology of normal rat subcutaneous loose connective tissues. The cross sections of normal rat subcutaneous connective tissues were immunostained by the antibodies against Sox10 (a-d), CD31 (a), ACTA2 (b), NG2 (c) and Tuj1 (d). Cell nuclei were stained by DAPI. Arrow, $Sox10^+$ cells. Scale bar, 10 μm.

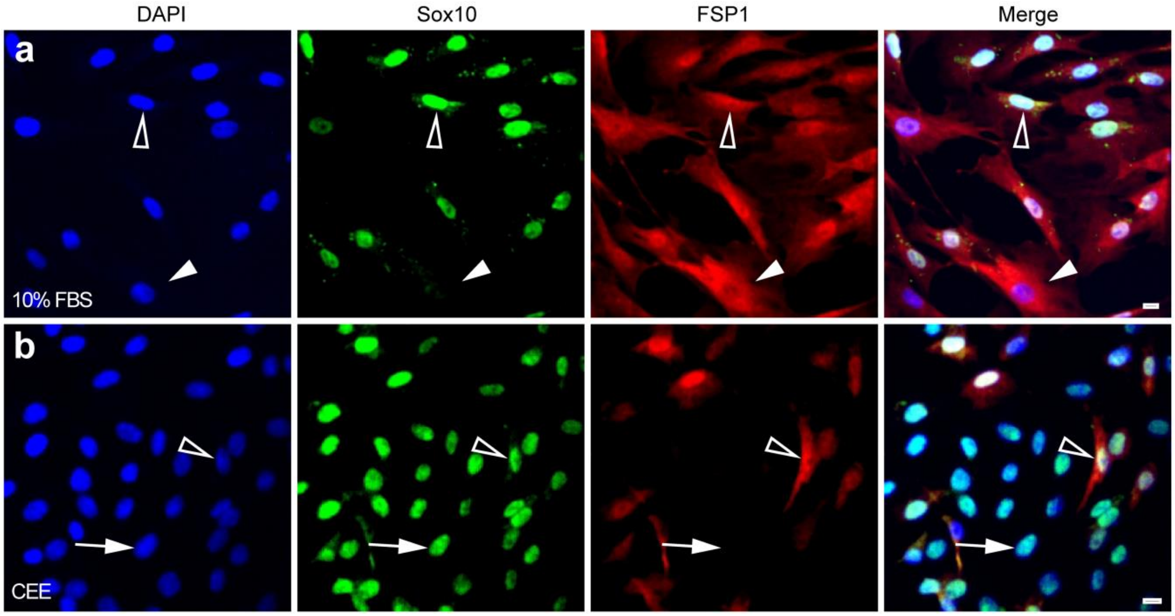

Figure S2. Immunostaining of primary cells *in vitro*. Primary cells from rat subcutaneous tissues cultured in the media of 10% FBS (a) and CEE (b) were immunostained by the antibodies against Sox10 and FSP1. Cell nuclei were stained by DAPI. Arrow, $Sox10^+$ cells. Arrowhead, $FSP1^+$ cells. Hollow arrowhead, $Sox10^+/FSP1^+$ cells. Scale bar, 10 μm.

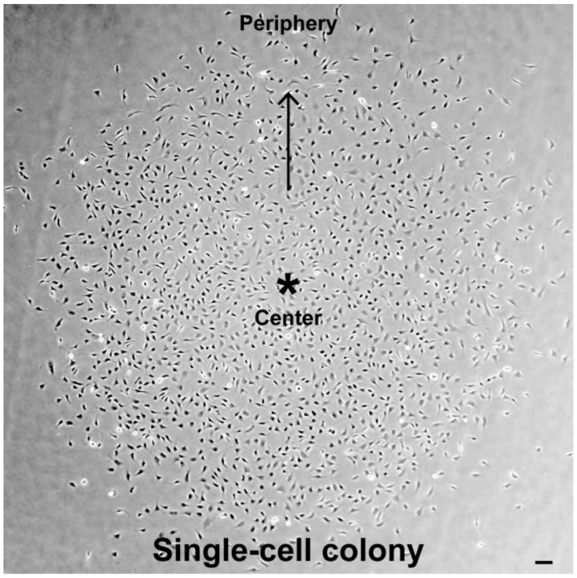

Figure S3. Phase contract image of a single-cell colony. Star indicates the center of the colony. Scale bar, 100 μm.

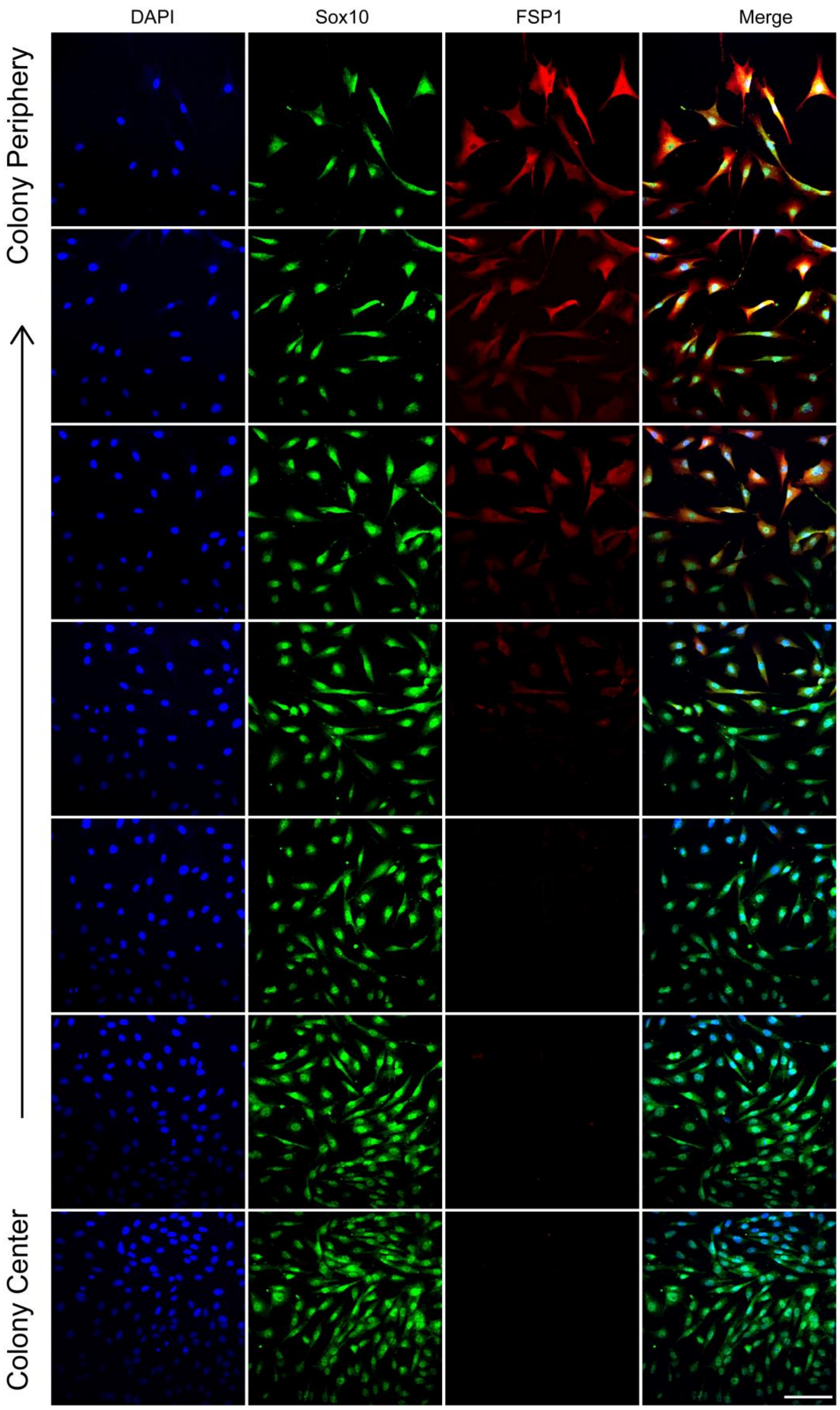

Figure S4. Immunostaining of single-cell colony *in vitro*. The cells of a single-cell colony were immunostained by the antibodies against Sox10 and FSP1. Scale bar, 100 μm.

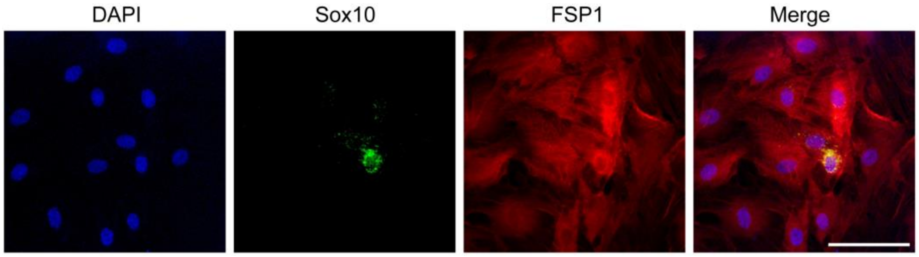

Figure S5. Immunostaining of differentiated fibroblasts *in vitro*. The cells of single-cell colonies after two passages in the medium with 10% FBS were immunostained by the antibodies against Sox10 and FSP1. Scale bar, 100 μm.

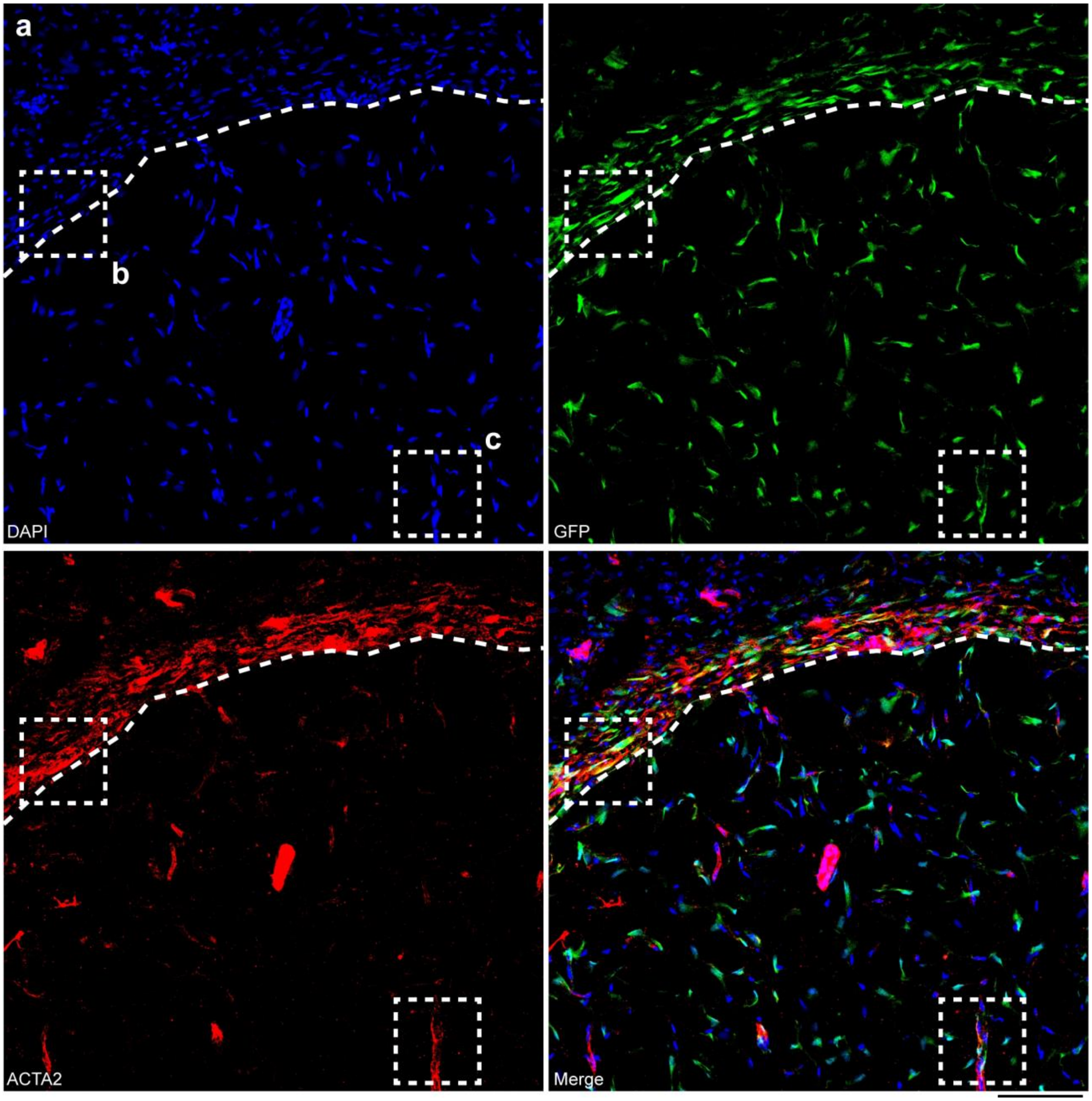

Figure S6. Immunohistology of Matrigel plug. (a) The cross section of the Matrigel plug with GFP$^+$ cells was immunostained by the antibody against ACTA2. Cell nuclei were stained by DAPI. The dashed curves outline the capsule layer around the Matrigel plug. The dashed squares indicate two representative regions in peripheral capsule layer (b) and central microvessels (c). Scale bar, 100 μm.

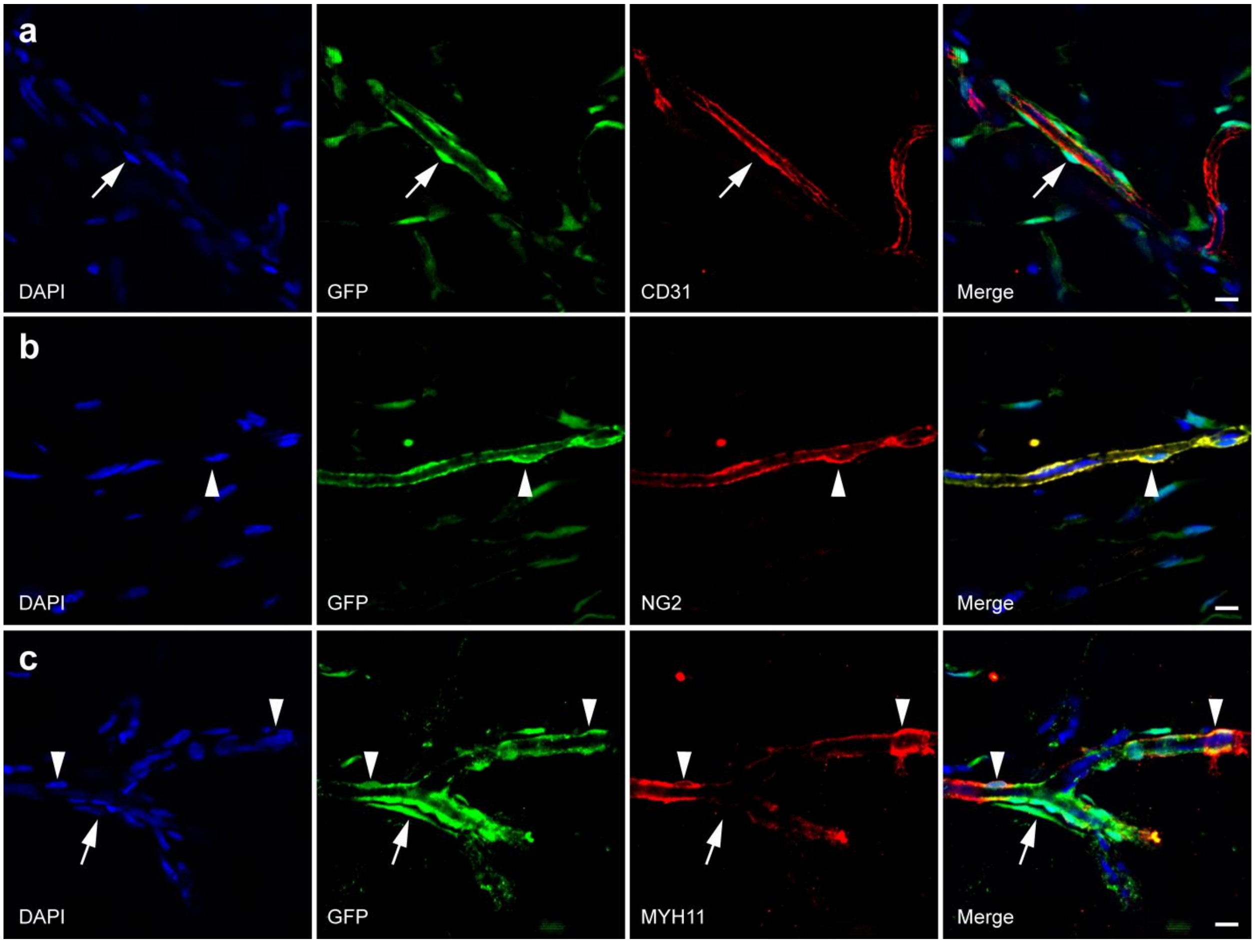

Figure S7. Immunohistological analysis of vascular markers of GFP$^+$ vessels. The cross sections of the Matrigel plug with GFP$^+$ cells were immunostained by the antibodies against CD31 (a), NG2 (b) and MYH11 (c). Cell nuclei were stained by DAPI. Arrow, GFP$^+$ cells. Arrowhead, double positive cells. Scale bar, 10 μm.

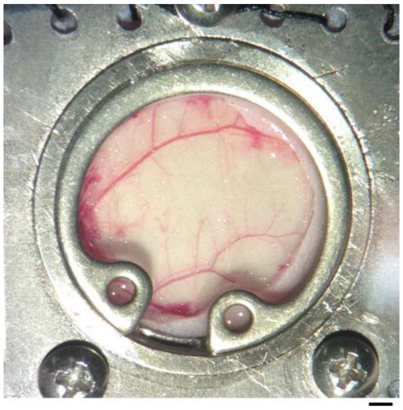

Figure S8. Illustration of mouse dorsal skinfold chamber. Scale bar, 1 mm.
